# Prevalence of Enterococcus Species in Various Clinical Samples and Their Antimicrobial Susceptibility Pattern

**DOI:** 10.7759/cureus.72836

**Published:** 2024-11-01

**Authors:** Manasi Mahajan, Ravindra Shinde, Geeta S Karande, Satish Patil

**Affiliations:** 1 Department of Microbiology, Krishna Vishwa Vidyapeeth (Deemed to be University), Karad, IND

**Keywords:** antibiotic resistance, antibiotic susceptibility, gram-positive bacteria, healthcare-associated infections (hais), kirby-bauer disc diffusion

## Abstract

Background and aim

Gram-positive enterococci, which are normally prevalent in the intestine as commensals, have become important pathogens that cause serious illnesses such as meningitis, bacteremia, and endocarditis, especially in people with impaired immune systems. Due to the presence of resistance genes such as van A, B, and C, which lead to multidrug resistance, the two prevalent species, *Enterococcus faecalis* and* Enterococcus faecium*, are causing increasing concerns. In order to effectively combat these dangerous infections, there is an urgent need for improved surveillance, infection control measures, and antimicrobial stewardship. Research on the genetic features of enterococci is still lacking in India. The purpose of this work is to assess the frequency of* Enterococcus* isolates from different clinical samples and to examine the patterns of antibiotic resistance in these isolates.

Materials and methods

The purpose of this cross-sectional observational study was to assess the prevalence of *Enterococcus* isolates from various clinical specimens. Based on information from previous studies, a sample size of 114 was chosen. Urine, blood, sputum, wound swabs, and sterile body fluids were all used for the isolation of bacteria. Through the use of biochemical tests such as bile esculin hydrolysis and salt tolerance, gram staining, and cultural traits, enterococci were identified. The Kirby-Bauer disc diffusion method was utilized to evaluate antibiotic susceptibility, with a specific focus on resistance patterns. The findings show that in order to address the increasing problems that these pathogens are posing in hospital settings, there is an urgent need for improved surveillance and efficient infection control strategies.

Results

The results of the investigation showed that different clinical specimens had different distributions of *Enterococcus* species. The majority of isolates (69.16%) came from urine samples, with blood samples making up the remaining 12.5%. The age group between 41 and 50 years old had the highest occurrence, with a notable male predominance. The majority of *Enterococcus* isolates (96.66%) came from inpatient settings, specifically from the critical care units (CCU) and intensive care units (ICU). Teicoplanin sensitivity was highest among the isolates (79.16%).

Conclusion

In our study, 120 *Enterococcus* isolates in all, mostly of* Enterococcus faecium* and *Enterococcus faecalis*, were isolated from different clinical specimens for our investigation. Teicoplanin showed the highest sensitivity among the isolates. The study of *Enterococcus* species prevalence and antimicrobial susceptibility is vital for informing infection control and treatment strategies, enhancing antibiotic stewardship, and guiding public health policies.

## Introduction

Enterococci are cocci that are gram-positive, known only as intestinal commensals with little significance, considered virtually as harmless bacteria [1]. Normally, it represents a component of the native microbiota of the intestinal tract, oral cavity, and genitourinary tract in humans and animals [2]. Nine genes have been identified and characterized that ascertain vancomycin resistance, namely, van A, B, C, D, E, G, L, M, and N [3]. The rise of multidrug-resistant (MDR) enterococcihas created a situation nearly as critical as the most severe cases of the pre-antibiotic era, as numerous MDR strains have acquired resistance to nearly all available antibiotics [4].

The evolution and development of the transpose's elements confer the "vancomycin" resistance within *Enterococcus *species [5]. Vancomycin resistance among *Enterococcus* prevalence of vancomycin-resistant *Enterococcus* (VRE) isolates represents a significant issue in much of the Western world, particularly in the United States. According to data from the National Nosocomial Infections Surveillance (NNIS), over 28% of nosocomial *Enterococcus* strains exhibit resistance to vancomycin [6].

In the Indian context, resistance to aminoglycosides in enterococcihas been addressed in a limited number of studies [7]. There is a significant lack of detailed information concerning the genetic basis of vancomycin resistance in *Enterococcus* isolates from clinical specimens collected at a tertiary care center with a special emphasis on vancomycin resistance in enterococci and its genetic basis.

They present a particular risk for infections associated with catheters and other implanted medical devices in critically ill patients, and they are also implicated in late-onset sepsis, pneumonia, and meningitis in neonates [8]. In the 2006-2007 report from the Centers for Disease Control and Prevention (CDC), enterococciaccount for approximately 12% of healthcare-associated infections (HAIs) and rank as the third most prevalent MDR pathogen contributing to these infections [9]. Since the initial report of the emergence of VRE reported in the United Kingdom and France, there has been a considerable increasing incidence of VRE reported across multiple countries, including Australia, Canada, Germany, Malaysia, Spain, and the United States [10]. The study aims to isolate and identify enterococci from various clinical samples using standard microbiological techniques. Following isolation, the isolates will be characterized to the species level. Additionally, the study will assess the antibiotic susceptibility patterns of these isolates.

## Materials and methods

Study design

This is a cross-sectional, observational study.

Study period

The study was conducted from November 2022 to November 2023.

Sample size

As per the study undertaken by Shah et al. [11] in the Department of Microbiology, Mumbai, and referring to their prevalence rate, the sample size was calculated using the formula n=4pq/l^2^. One hundred twenty clinical samples were collected and processed.

Data collection

Clinical samples from the Krishna Hospital and Medical Research Centre (KH & MRC) were collected and processed at the Department of Microbiology, Krishna Institute of Medical Sciences, Karad. 

Inclusion criteria 

Samples included urine, blood, sterile body fluids, wound swabs, sputum, and other specimens from patients with suspected infections admitted to inpatient and outpatient departments, encompassing all genders and age groups.

Exclusion criteria

Isolation ofenterococcifrom the same patients and from the same specimens will be excluded from the study to avoid the duplication of isolates.

Phenotypic characterization

The clinical samples were processed to identify and characterize *Enterococcus *species. Samples, including urine, blood, pus, and wound swabs, were cultured on various media such as nutrients, blood, and MacConkey agar. Identification involved gram staining, catalase testing, and biochemical assays like bile esculin hydrolysis, salt tolerance, and arginine dihydrolase test.* Enterococcus faecalis* and *Enterococcus faecium* were identified and tested for resistance to various antibiotics, including vancomycin, highlighting the importance of ongoing surveillance and effective infection control in managing antibiotic resistance in healthcare settings.

Antimicrobial susceptibility test

**Figure 1:**
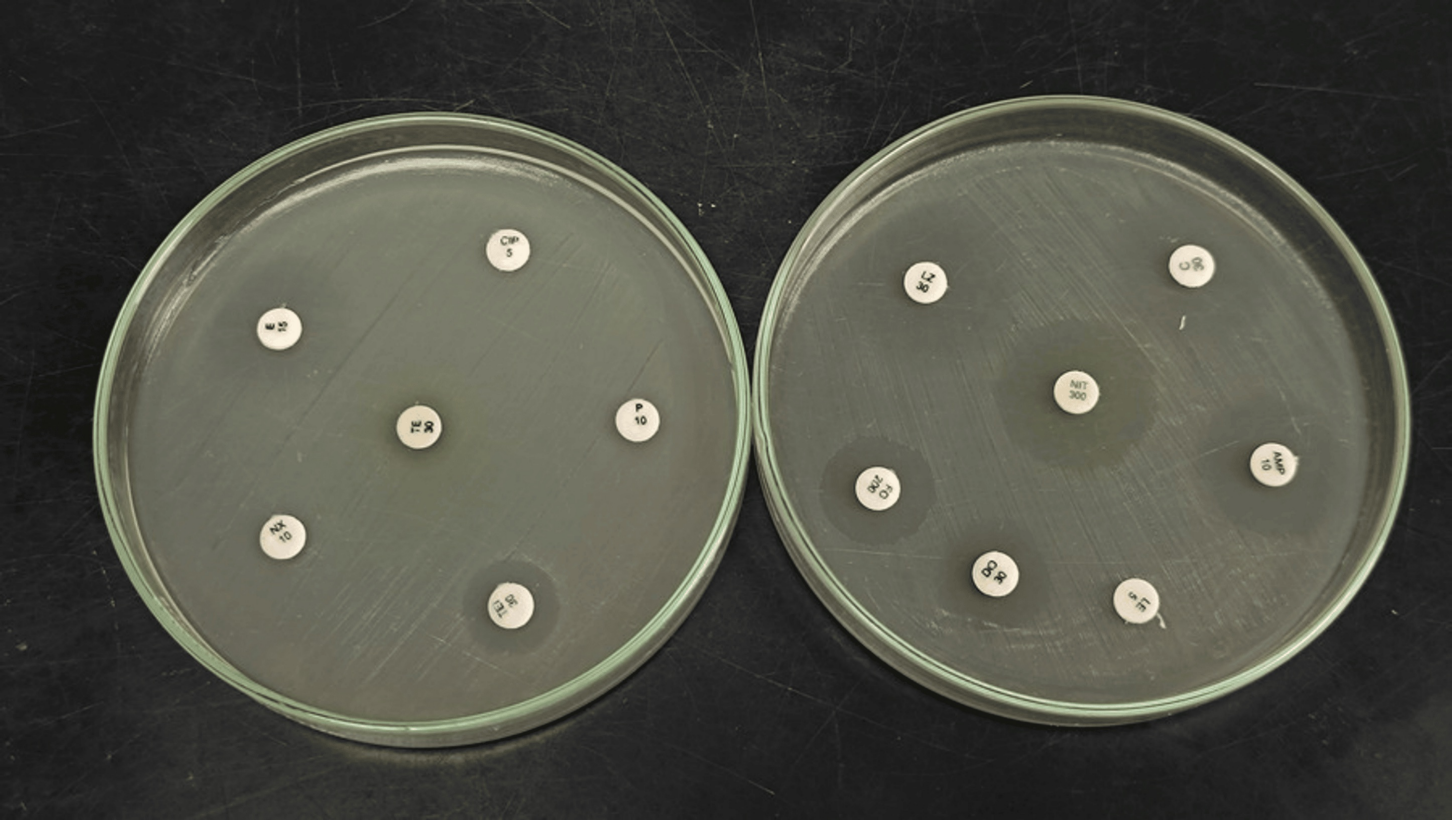
Antimicrobial susceptibility pattern The left image and right image show antibiotic susceptibility on the Petri dish (left) and Petri dish (right), with zones of inhibition indicating varying levels of resistance or susceptibility to various antibiotics.

Antibiotic susceptibility testing was conducted using the Kirby-Bauer disc diffusion method, according to the Clinical and Laboratory Standards Institute (CLSI) [12]. The bacterial inoculum of test and control was prepared to match a 0.5% McFarland turbidity standard. Then the bacterial inoculum was uniformly distributed onto the Mueller-Hinton agar plates. After drying, antibiotic discs were placed on the agar, and the plates were incubated at 37°C for 24 hours as seen in Figure 1. The susceptibility was tested against ampicillin (10 μg), penicillin (10 μg), nitrofurantoin (300 μg), teicoplanin (30 μg), erythromycin (15 μg), tetracycline (30 μg), doxycycline (30 μg), levofloxacin (5 μg), chloramphenicol (30 μg), linezolid (30 μg), ciprofloxacin (5 μg), fosfomycin (200 μg), and norfloxacin (10 μg). The control strain was *Enterococcus faecalis *29212.

Statistical analysis

Data were recorded in Microsoft Excel (Microsoft Corp., Redmond, WA, USA), and the analyzed results were further presented as numbers and percentages, and p-values were calculated from the chi-squared test using the GraphPad InStat program version 3.06, 32-bit for Windows (Insight Venture Management, LLC, New York, NY, USA). A significant relationship or difference is indicated by a p-value less than 0.05.

Ethical clearance

Consent was taken from all participants in this study. Approval was granted by the Institutional Ethics Committee of Krishna Institute of Medical Sciences, Krishna Vishwa Vidyapeeth (Deemed to be University) (approval number: 074/2021-2022). The authors affirm that this study adhered to ethical standards and received the necessary approval from the said committee.

## Results

Demographic data

Table 1 shows the distribution of *Enterococcus* spp. according to different age groups and genders. In the 0-10 age group, two isolates (1.66%) were obtained while in the 11-20 age group five isolates (4.16%). In the 21-30 age group, 20 isolates were obtained and nine isolates (7.5%) in the 31-40 age group. The 41-50 age group had 29 isolates (24.16%), and in the 51-60 age group, there were 15 isolates (12.5%). Among those aged 61-70 years, 24 isolates (20%) were reported, and 12 isolates (10%) were reported in individuals aged 71 years and older. In our area, males are more likely to work outside and have higher environmental exposure, which is why this study observed a higher infection rate among males.

**Table 1:** Age- and gender-wise distribution of Enterococcus species n: number; %: percentage

Age group (in years)	Male n (%)	Female n (%)	Total n (%)
0-10	0	2 (3.70)	2 (1.66)
11-20	2 (3.03)	3 (5.55)	5 (4.16)
21-30	9 (13.6)	15 (27.77)	24 (20)
31-40	5 (7.5)	4 (7.40)	9 (7.5)
41-50	20 (30.3)	9 (16.66)	29 (24.16)
51-60	10 (15.15)	5 (9.25)	15 (12.5)
61-70	12 (18.18)	12 (22.22)	24 (20)
>71	8 (12.12)	4 (7.40)	12 (10)
Total	66 (55)	54 (45)	120 (100)

Analysis of clinical samples

Table 2 shows the distribution of *Enterococcus* species according to different sample types. The majority of isolates were from urine samples, accounting for 83 isolates (69.16%), followed by blood samples with 15 isolates (12.5%), while pus swabs accounted for six isolates (5%), wound samples yielded four isolates (3.33%), and vaginal swabs and fluids contributed three isolates each (2.5%). Tissue bits and self-retaining catheter (SRC) tips/endotracheal tube (ETT) accounted for three isolates each (2.5%) with the sputum sample giving one isolate (0.85%).

**Table 2:** Distribution of Enterococcus species from various clinical specimens n: number; %: percentage; SRC: self-retaining catheter; ETT: endotracheal tube

Specimens	Number (n)	Percentage (%)
Urine	83	69.16
Blood	15	12.5
Pus swab	6	5
Wound	4	3.33
Vaginal swab	3	2.5
Fluids	2	1.66
Tissue bit	3	2.5
SRC tips/ETT	3	2.5
Sputum	1	0.85
Total	120	100

Table 3 shows that *Enterococcus faecium* was the most common species isolated (64, 53.33%), followed by *Enterococcus faecalis* (56, 46.67%).

**Table 3:** Speciation of Enterococcus isolates n: number; %: percentage

Species	No. of isolates (n)	Percentage (%)
Enterococcus faecium	64	53.33
Enterococcus faecalis	56	46.67
Total	120	100

Table 4 shows the distribution of *Enterococcus *species in various inpatient department (IPD)/outpatient department (OPD). Maximum isolates of *Enterococcus* species were from IPD, in which 62 (51.67) were *Enterococcus faecium* and 54 (45.01) *Enterococcus faecalis*. Four enterococci species were isolated from OPD, in which two (1.66) each were *Enterococcus faecium* and *Enterococcus faecalis*. 

**Table 4:** Distribution of Enterococcus species from various clinical specimens received from IPD and OPD IPD: inpatient department; OPD: outpatient department; n: number; %: percentage

Clinical sample	IPD		OPD		Total n (%)
	*Enterococcus faecium* n (%)	*Enterococcus faecalis* n (%)	*Enterococcus faecium* n (%)	*Enterococcus faecalis* n (%)	
Urine	44 (36.66)	38 (31.66)	0	1 (0.83)	83 (69.15)
Blood	9 (7.5)	6 (5)	0	0	15 (12.5)
Pus swab	2 (1.66)	2 (1.66)	1 (0.83)	1 (0.83)	6 (4.98)
Vaginal swab	0	3 (2.5)	0	0	3 (2.5)
Wound	3 (2.5)	1 (0.83)	1 (0.83)	0	4 (4.23)
Fluids	1 (0.83)	1 (0.83)	0	0	2 (1.66)
Tissue bit	1 (0.83)	1 (0.83)	0	0	2 (1.66)
Tips/tubes	2 (1.66)	1 (0.83)	0	0	3 (2.49)
Sputum	0	1 (0.83)	0	0	1 (0.83)
Total	62 (51.67)	54 (45.01)	2 (1.66)	2 (1.66)	120 (100)

Sampling distribution and pattern of resistance

Table 5 shows the isolation of *Enterococcus* spp. from the patient sample, demonstrating a higher frequency among indoor patients, with 116 isolates (96.66%) compared to outdoor patients, where only four isolates (3.34%) were found. 

**Table 5:** Department-wise distribution of inpatient and outpatient among the Enterococcus species n: number; %: percentage

Department	Number (n)	Percentage (%)
Inpatient	116	96.66
Outpatient	4	3.34
Total	120	100

Table 6 shows the distribution of samples received from various indoor sections of the hospital indicating that the highest number of isolates was from the intensive care unit (ICU) patients, accounting for 39 (32.5%) of the total, followed by the coronary care unit (CCU) with 34 (28.33%), medicine 12 (10%), neurology 13 (10.83%), gynecology and obstetrics and gynecology (OBGYN) four each (3.33%), surgery four (3.33%), neonatal intensive care unit (NICU), oncology, ortho, and cath lab two each (1.66%), and cardiovascular and thoracic surgery (CVTS) and ear, nose, and throat (ENT) one each (0.86%).

**Table 6:** Distribution of samples obtained from various indoor sections of the hospital n: number; %: percentage; ICU: intensive care unit; CCU: coronary care unit; OBGYN: obstetrics and gynecology; NICU: neonatal intensive care unit; Cath lab: cardiac catheterization lab; CVTS: cardiovascular and thoracic surgery; ENT: ear, nose, and throat; IPD: inpatient department

IPD wards	No. of samples (n)	Percentage (%)
ICU	39	32.5
CCU	34	28.33
Medicine	12	10
Neurology	13	10.83
Gynecology and OBGYN	4	3.33
Surgery	4	3.33
NICU	2	1.66
Oncology	2	1.66
Ortho	2	1.66
Cath lab	2	1.66
CVTS	1	0.86
ENT	1	0.86
Total	116	96.68

Table 7 shows the distribution of samples received from various outdoor sections of the hospital indicating the number of isolates from orthopedics, medicine, surgery, and OBGYN, each accounting for one (0.83%) of the total. 

**Table 7:** Distribution of samples obtained from various outdoor sections of the hospital n: number; %: percentage; OBGYN: obstetrics and gynecology

OPD wards	Sample (n)	Percentage (%)
Orthopedic	1	0.83
Medicine	1	0.83
Surgery	1	0.83
OBGYN	1	0.83
Total	4	3.32

Table 8 shows that the different bacterial isolates exhibit varying patterns of resistance to antimicrobial agents. *Enterococcus* spp. showed the highest sensitivity to teicoplanin (79.16%). Conversely, the highest resistance was observed to erythromycin (100%), followed by penicillin, ciprofloxacin, and norfloxacin (97.5% each) and levofloxacin (95.83%). 

**Table 8:** Antibiotic susceptibility pattern of Enterococcus species isolated from various samples Chi-squared test: 541.42; p<0.0001: statistically significant; n: number; %: percentage

Antibiotic	Sensitive		Resistant	
	No. of isolates (n)	Percentage (%)	No. of isolates (n)	Percentage (%)
Ampicillin	47	39.16	73	60.83
Penicillin	3	2.5	117	97.5
Teicoplanin	95	79.16	25	20.83
Erythromycin	0	0	120	100
Tetracycline	4	3.33	116	96.66
Doxycycline	21	17.5	99	82.5
Levofloxacin	5	4.16	115	95.83
Chloramphenicol	56	46.66	64	53.33
Linezolid	42	35	78	65
Nitrofurantoin	48	40	72	60
Ciprofloxacin	3	2.5	117	97.5
Fosfomycin	79	65.83	41	34.16
Norfloxacin	3	2.5	117	97.5

## Discussion

In the current investigation, the majority of the isolates (83, 69.16%) originated from urine, with blood accounting for 12.5% (n=15), pus swab 5% (n=6), and wound 3.33% (n=4). Other sources such as vaginal swabs, tissue bits, fluids, SRC tip/ETT, and sputum constituted smaller proportions ranging from 2.5% to 0.85%. These findings are consistent with earlier studies. Mathew [13] found a predominant presence of *Enterococcus* species in urine (96, 61.5%), followed by blood (29, 18.6%) and pus (25, 16%). Similarly, Oberoi and Aggarwal [14] reported a significant proportion of isolates from urine (58, 76.31%) and a minor fraction from blood (6, 7.89%). Praharaj et al. [15] also highlighted a substantial isolation rate from urine (217, 59.1%). Mishra et al. [16] recorded the highest isolation rate in urine (150, 75%) and blood (26, 13%), with lower rates observed in pus (18, 9%) and other sources (6, 3%).

The current study demonstrates that of 120 isolates, the majority of the isolates were in the age group of 41-50 years (29, 24.16%). This is somewhat similar to Ravi et al. [17] showing the majority of isolates in the age group 31-50 years (74, 37%). In the present study, the majority of the isolates were males (66, 55%) than females (54, 45%) which were similar to the results of the study by Golia et al. [18] who reported 61 (61%) males and 39 (39%) females. Chanda et al. [19] found 34 (68%) males and 16 (32%) females.

The data from this study indicate that the majority of isolates (116, 96.66%) were obtained from IPD, with a small fraction (4, 3.34%) from OPD. This distribution is consistent with findings reported in other studies. Gulzar and Saikumar [20] observed 13792 (78.8%) of isolates from IPD and 3720 (21.2%) from OPD. Sharma et al. [21] noted 35 (48.61%) from IPD and 37 (51.38%) from OPD which were in contrast with the present study. Yadav et al. [22] reported 189 (94.5%) from IPD and 11 (5.5%) from OPD. This discrepancy may be ascribed to the higher exposure of patients in IPD settings to *Enterococcus*, which is frequently encountered in hospital environment. Recent investigations have established a distinct distribution of *Enterococcus* species, specifically *E. faecium* and *E. faecalis.*

In our study, the prevalence of *E. faecium* was 64 (53.33%), while *E. faecalis* accounted for 56 (46.67%). The present study is similar to Banerjee and Anupurba [23] reporting a prevalence rate of 148 (40.54%) for *E. faecium *and 169 (46.30%) for *E. faecalis*. Abamecha et al. [24] found slightly lower rates with *E. faecium* (41, 35.1%) and *E. faecalis* (34, 29.5%). Shokouhi et al. [25] identified a significant predominance of *E. faecium* (92, 91.1%), contrasting with *E. faecalis* (9, 8.9%). Rana and Sande [26] reported lower rates of *E. faecium *(7, 11.8%) and higher rates of *E. faecalis *(48, 81.3%).

In the present study, the majority of enterococcal infections were detected from IPD patients and more frequently observed in patients in the ICU (39, 32.5%) followed by the CCU with 34 (28.33%), medicine 12 (10%), neurology 13 (10.83%), gynecology and OBGYN four each (3.33%), surgery four (3.33%), and NICU two (1.66%). The maximum number of enterococcal infections was detected from OPD patients with orthopedics, medicine, surgery, and OBGYN, each accounting for one (0.83%) of the total. Similarly, Sahu et al. [27] have reported that the predominant proportion of enterococcal infection was detected from IPD patients and more frequently observed in patients in the ICU (49, 32.3%) followed by the surgical unit (37, 24.3%) and the medicine unit (30, 19.7%) and the maximum number of *Enterococcus* infections was detected from OPD patients (36, 23.7%).

There are several important limitations that restrict the study of *Enterococcus *species prevalence and antimicrobial susceptibility. Results that are not sufficiently diverse in terms of demographics or sample sizes may not be sufficiently generalizable. Inconsistencies could be brought about by variations in laboratory procedures, and strains that are resistant to multiple drugs can make susceptibility assessments more difficult to make. Moreover, variations in infection management strategies and temporal changes in resistance patterns can affect the validity of results, emphasizing the necessity of cautious interpretation and ongoing study in this field.

## Conclusions

In the current study, *Enterococcus *is a significant nosocomial pathogen causing a variety of hospital-acquired infections and also community-acquired infections, contributing significantly to the patient's morbidity and mortality. In the present study, a total of 120 *Enterococcus *isolates derived from various clinical specimens were isolated with *Enterococcus faecium* and *Enterococcus faecalis* as the predominant species. They indicated resistance to multiple antibiotics like penicillin, erythromycin, levofloxacin, and norfloxacin. Recent advances in the management of *Enterococcus* infections have introduced several new drugs and treatment strategies, particularly in response to growing antibiotic resistance.

## References

[1] Marothi YA, Agnihotri H, Dubey D (2005). Enterococcal resistance - an overview. Indian J Med Microbiol.

[2] Uttley AH, George RC, Naidoo J (1989). High-level vancomycin-resistant enterococci causing hospital infections. Epidemiol Infect.

[3] Kafil HS, Asgharzadeh M (2014). Vancomycin-resistant enteroccus faecium and enterococcus faecalis isolated from education hospital of iran. Maedica (Bucur).

[4] Arias CA, Murray BE (2009). Antibiotic-resistant bugs in the 21st century — a clinical super-challenge. N Engl J Med.

[5] Davis E, Hicks L, Ali I (2020). Epidemiology of vancomycin-resistant Enterococcus faecium and Enterococcus faecalis colonization in nursing facilities. Open Forum Infect Dis.

[6] (2003). National Nosocomial Infections Surveillance (NNIS) system report, data summary from January 1992 through June 2003, issued August 2003. Am J Infect Control.

[7] Agarwal J, Kalyan R, Singh M (2009). High-level aminoglycoside resistance and beta-lactamase production in enterococci at a tertiary care hospital in India. Jpn J Infect Dis.

[8] Arias CA, Contreras GA, Murray BE (2010). Management of multidrug-resistant enterococcal infections. Clin Microbiol Infect.

[9] Hidron AI, Edwards JR, Patel J, Horan TC, Sievert DM, Pollock DA, Fridkin SK (2008). NHSN annual update: antimicrobial-resistant pathogens associated with healthcare-associated infections: annual summary of data reported to the National Healthcare Safety Network at the Centers for Disease Control and Prevention, 2006-2007. Infect Control Hosp Epidemiol.

[10] Cetinkaya Y, Falk P, Mayhall CG (2000). Vancomycin-resistant enterococci. Clin Microbiol Rev.

[11] Shah S, Rampal R, Thakkar P, Poojary S, Ladi S (2022). The prevalence and antimicrobial susceptibility pattern of gram-positive pathogens: three-year study at a tertiary care hospital in Mumbai, India. J Lab Physicians.

[12] (2022). Performance standards for antimicrobial susceptibility testing. https://www.standards-global.com/wp-content/uploads/pdfs/preview/2247002.

[13] Mathew SK (2018). A profile of vancomycin-resistant enterococcal infections and a comparison of resistance detection methods. Indian J Microbiol Res.

[14] Oberoi L, Aggarwal A (2010). Multidrug resistant enterococci in a rural tertiary care hospital-a cause of concern. JK Science.

[15] Praharaj I, Sujatha S, Parija SC (2013). Phenotypic & genotypic characterization of vancomycin resistant Enterococcus isolates from clinical specimens. Indian J Med Res.

[16] Mishra M, Sharma A, Chauhan N, Mishra S (2022). Prevalence and antibiotic resistance pattern of isolated Enterococcus by standard techniques. Int J Health Sci Res.

[17] Ravi D, Rompicherla V, Govindan G, Shanmugam P (2016). Speciation of enterococcal isolates in a tertiary care hospital and molecular characterisation of vancomycin resistant enterococci (VRE). Indian J Microbiol Res.

[18] Golia S, Nirmala AR, Kamath AS (2014). Isolation and speciation of enterococci from various clinical samples and their antimicrobial susceptibility pattern with special reference to high level aminoglycoside resistance. Int J Med Res Health Sci.

[19] Chanda S, Suryawanshi R, Megha GK, Khutade K (2022). Phenotypic characterization and antibiotic susceptibility pattern of clinical isolates of enterococci with special emphasis on vancomycin resistance. Int J Sci Healthc Res.

[20] Gulzar B, Saikumar C (2021). Study on the prevalance, identification and antibiogram of Enterococci isolated from the heterogenous clinical samples in a tertiary care hospital. J Pharm Res Int.

[21] Sharma HK, Shakir SM, Sharma V (2023). Vancomycin resistant enterococci isolated from urinary tract infections in tertiary care hospital. J Cardiovasc Dis Res.

[22] Yadav G, Thakuria B, Madan M, Agwan V, Pandey A (2017). Linezolid and vancomycin resistant enterococci: a therapeutic problem. J Clin Diagn Res.

[23] Banerjee T, Anupurba S (2016). Risk factors associated with fluoroquinolone-resistant enterococcal urinary tract infections in a tertiary care university hospital in North India. Indian J Med Res.

[24] Abamecha A, Wondafrash B, Abdissa A (2015). Antimicrobial resistance profile of Enterococcus species isolated from intestinal tracts of hospitalized patients in Jimma, Ethiopia. BMC Res Notes.

[25] Shokouhi S, Darazam IA, Javadi A, Rouhani M, Ghasemnejad M (2017). Genotypic characterization of vancomycin-resistant Enterococcus spp. in tertiary center, Iran. Infect Disord Drug Targets.

[26] Rana D, Sande S (2020). Study of prevalence and antimicrobial susceptibility pattern of enterococci isolated from clinically relevant samples with special reference to high level aminoglycoside resistance (HLAR) in a rural tertiary care hospital. J Evol Med Dent Sci.

[27] Sahu LS, Dash M, Paty BP, Purohit GK, Chayan N (2015). Enterococcal infections and antimicrobial resistance in a tertiary care hospital, Eastern India. Afro-Egy J Infect Endem Dis.

